# *ANO7* African-ancestral genomic diversity and advanced prostate cancer

**DOI:** 10.1038/s41391-023-00722-x

**Published:** 2023-09-25

**Authors:** Jue Jiang, Pamela X. Y. Soh, Shingai B. A. Mutambirwa, M. S. Riana Bornman, Christopher A. Haiman, Vanessa M. Hayes, Weerachai Jaratlerdsiri

**Affiliations:** 1 Ancestry and Health Genomics Laboratory, Charles Perkins Centre, School of Medical Sciences, Faculty of Medicine and Health, University of Sydney, Camperdown, NSW Australia; 2 Department of Urology, Sefako Makgatho Health Science University, Dr George Mukhari Academic Hospital, Medunsa, South Africa; 3 School of Health Systems & Public Health, University of Pretoria, Pretoria, South Africa; 4 Center for Genetic Epidemiology, Department of Population and Public Health Sciences, Keck School of Medicine, University of Southern California, Los Angeles, CA USA; 5 Manchester Cancer Research Centre, University of Manchester, Manchester, UK

**Keywords:** Cancer genetics, Predictive markers

## Abstract

**Background:**

Prostate cancer (PCa) is a significant health burden for African men, with mortality rates more than double global averages. The prostate specific Anoctamin 7 (*ANO7*) gene linked with poor patient outcomes has recently been identified as the target for an African-specific protein-truncating PCa-risk allele.

**Methods:**

Here we determined the role of *ANO7* in a study of 889 men from southern Africa, leveraging exomic genotyping array PCa case-control data (*n* = 780, 17 *ANO7* alleles) and deep sequenced whole genome data for germline and tumour *ANO7* interrogation (*n* = 109), while providing clinicopathologically matched European-derived sequence data comparative analyses (*n* = 57). Associated predicted deleterious variants (PDVs) were further assessed for impact using computational protein structure analysis.

**Results:**

Notably rare in European patients, we found the common African PDV p.Ile740Leu (rs74804606) to be associated with PCa risk in our case-control analysis (Wilcoxon rank-sum test, false discovery rate/FDR = 0.03), while sequencing revealed co-occurrence with the recently reported African-specific deleterious risk variant p.Ser914* (rs60985508). Additional findings included a novel protein-truncating African-specific frameshift variant p.Asp789Leu, African-relevant PDVs associated with altered protein structure at Ca^2+^ binding sites, early-onset PCa associated with PDVs and germline structural variants in Africans (Linear regression models, −6.42 years, 95% CI = −10.68 to −2.16, *P*-value = 0.003) and *ANO7* as an inter-chromosomal PCa-related gene fusion partner in African derived tumours.

**Conclusions:**

Here we provide not only validation for *ANO7* as an African-relevant protein-altering PCa-risk locus, but additional evidence for a role of inherited and acquired *ANO7* variance in the observed phenotypic heterogeneity and African-ancestral health disparity.

## Introduction

Prostate cancer (PCa) is a significant health burden globally with mortality rates that vary dramatically by ethnicity [1, 2]. Being of African ancestry is a significant risk factor for aggressive presentation and associated mortality. Within the United States, African American men have a higher lifetime risk of dying from PCa [1] and a significantly higher mortality rate than men of European ancestry after adjusting for age, income and other factors [3]. PCa mortality rates are double the global averages in Sub-Saharan Africa, 2.7-fold greater in southern Africa compared to the United States [2]. Combined with substantial PCa heritability [4], a genomic study including men across the diverse spectrum of African ancestries provides an underappreciated opportunity to identify contributing genetic factors to PCa associated health disparity.

Anoctamin 7 (*ANO7*), also called *TMEM16G*, codes for a member of the anoctamin family which has been reported to be correlated with cancer progression [5]. The original name ‘New Gene Expressed in Prostate’ (*NGEP*) highlights the almost exclusive expression of *ANO7* in prostate epithelial cells [6]. While the function of *ANO7* in the prostate remains unknown, this transmembrane protein is suggested to be dependent on calcium (Ca^2+^) as a potential calcium-activated chloride channel (CACC) or a Ca^2+^-dependent phospholipid scramblase (PLS) [7]. *ANO7* tissue expression has been associated with PCa outcomes, with contradictory studies linking decreased [8–10], or increased [11] expression with aggressive disease. The latter study linking genotypes with expression suggests the contribution of genetic ancestry (Iranian and German *versus* Finnish) as contributing factors for the observed disparity. As a candidate PCa susceptibility gene, differences in associated risk alleles have been identified between ancestries. Most recently, an *ANO7* stop-gain variant p.Ser914* (rs60985508) has shown a significant association with PCa in men of African ancestry [12]. Conversely, the significance of four European-specific (85,554 cases, 91,972 controls) PCa-risk variants rs77559646, rs2074840, p.Ala759Thr (rs76832527) and p.Glu226Lys/p.Glu226* (rs77482050) were excluded from the analyses of men of African (10 368 cases, 10,986 controls) and East Asian (8611 cases, 18,809 controls) or Hispanic ancestries (2714 cases, 5 239 controls) [13]. Additionally, p.Arg30Gly/p.Arg30* (rs148609049) has been associated with reduced survival rates (1 627 Finnish cases) [11], rs77559646 with improved progression-free survival (110 and 98 Finnish cases) [14], and rs62187431 with lower risk for biochemical recurrence (638 Asian cases) [15].

Taken together, the literature suggests a link among genetic ancestry, the spectrum of *ANO7* variation, and PCa risk and/or disease outcome. Observing (as of 1 March 2023) a notable lack of pathogenic *ANO7* germline variants reported in ClinVar [16], we sought in this study to determine if germline and/or acquired variants within *ANO7* are contributing to aggressive PCa presentation in southern Africa. Here we interrogate *ANO7* germline and tumour genome sequencing (*n* = 166), as well as array-based genotype data (*n* = 780), providing the first insights for the relevance of *ANO7* and aggressive PCa presentation within the genetically diverse southern African population identifier.

## Subjects and methods

### Subjects and whole genome sequencing

Blood and tumour samples were collected from 166 patients diagnosed with PCa from South Africa (n = 113) and Australia (*n* = 53), with a bias towards high-risk cases (79.5% Gleason score $\ge$4 + 3, Supplementary Table S1). The samples underwent deep whole genome sequencing (WGS) using a single technical and Hg38 referenced variant calling and annotation pipeline, as previously described [17]. Ancestry inference was computed using fastSTRUCTURE population analysis with 7 472 833 germline single-nucleotide variants (SNVs). Of the 166 patients, 109 were categorised as African (all South African) having $\ge$98% African-ancestral fraction, and 57 as European (53 Australian, 4 South African) allowing up to 3% African ancestral and 26% Asian contributions [17]. Germline and somatic variants were selected in *ANO7* gene (GRCh38 assembly, allowing 1 kb extension at upstream and downstream) and including besides SNVs, also small insertion-deletions (indels) and larger structural variations (SVs).

### Exome array case-control study

The investigation of African-related PCa causal variants was conducted on a case-control study of 798 South Africans. Genotyping was conducted on the Infinium HumanExome-12 v1.0 BeadChip array (Illumina, California, United States). Subjects were filtered for admixture according to a principal component analysis (PCA) of the ancestry of subjects and were also filtered for relatedness (supplementary methods). Samples that passed quality control (*n* = 780; Table 1) included 473 cases (age median 71, range 49–102) and 307 controls (age median 70, range 45–99). Cases and controls are of similar age distribution (Wilcoxon test, *P*-value = 0.49). From 54 markers across the *ANO7* gene region, 17 single-nucleotide polymorphisms were represented within our study cohort (Supplementary Table S2).

**Table 1:** MAFs of ANO7 predicted deleterious variants (PDV) in WGS data and exome array data and population data.

		MAF in WGS data (%)			MAF in exome array data (%)				Online population data (%, ALFA)^a^		
rsID	Protein change (NP001001891)	African (109)	European (57)	PDV status (WGS)	Southern African cases (*n* = 473)	Southern African controls (*n* = 307)	PDV status (exome)	PDV consensus	African	European	Previous studies
rs144166359	p.Gly242Arg	0.46	0	Yes	0.42	0	Yes	Yes	0.1	0	No previous studies
rs201506858	p.Arg336His	0^a^	0	No	0.11	0.33	Yes	No	0.06	<0.01	No previous studies
rs111978925	p.Ala360Val	0	0	No	0.11	0	Yes	No	0.67	0.01	No previous studies
rs145388383	p.Cys397Tyr	0	0	No	0.11	0.65	Yes	No	0.28	<0.01	No previous studies
rs147670958	p.Tyr440Asn	1.83	0	Yes	1.16	0.82	Yes	Yes	0.59	0.01	No previous studies
rs145157097	p.Arg465Trp	0	0.88	Yes	0	0	No	No	0.03	0.16	No previous studies
rs887541003	p.Ala470Val	1.38	0	Yes	0	0	No	No	0	0.02	No previous studies
rs111934267	p.Arg578Cys	1.83	0	Yes	3.7	1.95	Yes	Yes	1.33	0.01	No previous studies
rs139066448	p.Ala632Val	0.46	0	Yes	0.11	0	Yes	Yes	0.6	0.01	No previous studies
Novel	p.Leu734Pro	0.46	0	Yes	0	0	No	No	0	0	No previous studies
rs74804606	p.Ile740Leu	16.97	0.88	Yes	21.56	15.36	Yes	Yes	15.62	0.19	Associated with the estrone/androstenedione ratio [40]
rs773052325	p.Ala744Gly	0.46	0	Yes	0	0	No	No	0	0	No previous studies
rs76832527	p.Ala759Thr	0	16.67	Yes	0.11	0.16	Yes	Yes	6.13	17.25	PCa, Asian population [15]; PCa, European population [31]; PCa, significant results in multiethnic analysis and European population, not in African-only population [13]; PCa and BPH, UK Biobank [32]
rs527323541	p.Asp789Val	2.29	0	Yes	0	0	No	No	0	0	No previous studies
Novel	p.Asp789Leu (frameshift)	2.29	0	Yes	0	0	No	No	0	0	No previous studies
rs60985508	p.Ser914^a^ (stop-gain)	35.78	0.88	Yes	0	0	No	No	28.49	0.4	African-specific PCa risk allele [12]

Variants with MAF = 0 are not identified in that group.

^a^ALFA: Derived from Allele Frequency Aggregator (ALFA, [22]).

### Annotation of short variants

The annotation of short variants (further outlined in supplementary methods) identified in both WGS and exome array was processed with the online tool SNPnexus (https://www.snp-nexus.org/v4/) [18]. SNPnexus provides multiple tools and datasets for annotation, including Sorting Intolerant From Tolerant (SIFT, Jan 2019 updated) [19], Polymorphism Phenotype (PolyPhen, Jan 2019 updated) [20], and cancer genome interpreter (CGI) [21]. Predicted deleterious variants (PDVs) were defined as variants with SIFT scores under 0.05 or PolyPhen scores greater than 0.446 or causing stop/gain or frameshift on the main transcript of *ANO7* ENST00000274979. Other variants included benign, tolerated or structural variants. Minor allele frequencies (MAF) of PDVs in African and European populations were obtained from the online Allele Frequency Aggregator (ALFA) [22].

### Sequence analysis

Sequence and phylogenetic analyses were conducted using MEGA (v11) [23] on 45 unique amino acid sequences of the *ANO7* transcript ENST00000274979. Sequences were aligned by MUSCLE [24] in MEGA and the best protein model was estimated by PhyML (v 3.0) [25], as detailed in supplementary methods. We used the neighbour-joining statistical method and bootstrap values equal to 1000 to construct a phylogenetic tree, which was only assessed for groupings due to low branch support.

### Statistical analysis

Significant thresholds were set as 0.05 for *P*-value and false discovery rate (FDR) corrected by Rstatix package (v 0.7.0) [26] if multiple tests were conducted for each variant. The same version of R (v 4.1.3; R Core Team, 2022) was used throughout the study.

Correlations between variants were tested using Spearman’s rank correlation coefficient (ρ) from Stats package in R, which assumes no frequency distribution. Haplotype block analysis used Haploview v4.1 [27]. Associations between age at diagnosis and selected *ANO7* variants were investigated with linear regression models using Stats package in R. African patients with age available (*n* = 108, one unavailable) were tested for carrying $\ge$3 selected variants. The best model was selected by the fitness of the model estimated by Akaike’s Information Criterion (AIC) in stepwise selection. PDV prevalence in ethnic groups was compared using logistic regression models from Stats package in R (supplementary methods). Genotypes identified in exome array data were compared between cases and controls using non-parametric Wilcoxon rank-sum test which fits non-normal distributed data.

### Prediction of protein structures and pores

The protein structure of amino acid sequences was predicted using the RaptorX Structure Prediction online server [28] which predicts protein structures by aligning the given sequence to known structures and uses convolutional neural networks (CNN) for a high quality contact map. The predicted structure was used for pore prediction by MOLE online tool [29] with default settings at 13 Å and 0.8 Å for the probe radius and interior threshold, respectively. MOLE identifies possible channels and merges them to pores with estimated physicochemical properties, such as hydropathy, radii, and bottleneck. The pore prediction of each protein was conducted over 20 times to achieve reproducible results that defined pores within the same group of helices more than three times. Approximately 2 to 3 distinctive pores were identified per protein structure.

## Results

### *ANO7* ancestral diversity

**Fig. 1:**
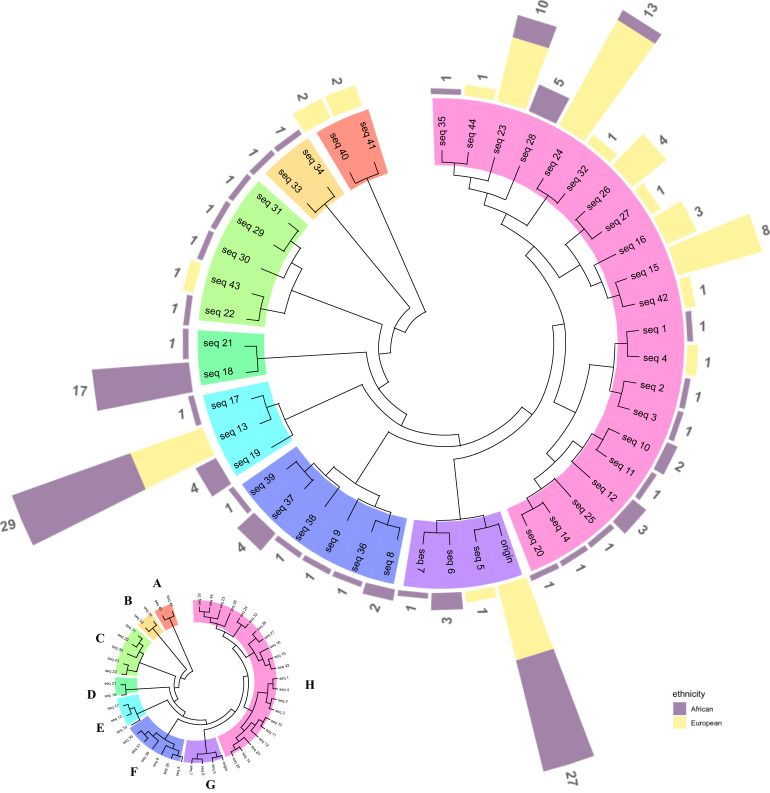
Phylogenetic inference of 45 unique germline sequences of *ANO7* gene (ENST00000274979 as a transcript reference). The inner circle shows the phylogenetic tree with colours representing eight groups A–H labelled in the bottom left plot. The averaged pairwise genetic distance between groups were ranged from 2.15e−3 to 0.11. The outer layer of the circle shows the number of samples sharing the same sequence. Numbers are for the count of samples for each sequence and coloured bars show the proportion of ethnicities for each sequence adjusted by the total count of samples in respective ethnic group (*n* = 109 Africans and *n* = 57 Europeans). Purple indicates Africans and yellow indicates Europeans.

A total of 809 germline variants were reported within the *ANO7* gene region for 166 WGS genomes, with as expected [17, 30], greater numbers observed for Africans over Europeans (median 125 vs 110). Exhibiting 45 unique amino acid sequences of *ANO7* containing germline missenses, with median pairwise genetic distance 0.003 (range, 0.001–0.005). The number of African-specific sequences were more than twice of the Europeans (29 *vs* 12), with African patients exhibiting three times as many individually unique African-specific sequences as Europeans (21 vs 7). Phylogenetic analysis divided the sequences into eight groups (Fig. 1) including three African-specific (Groups B, D and F) and one European-specific (Group A).

### *ANO7* germline predicted deleterious variants (PDVs)

Of the 13 germline variants identified in 166 WGS genomes annotated as PDVs (Table 1, Supplementary Fig. S1), nine were African-specific, including p.Leu734Pro and p.Asp789Leu novel to this study, while p.Ala744Gly (rs773052325) has previously been reported in a single East Asian (Supplementary Fig. S1). Known PCa variants included the European-exclusive PDV p.Ala759Thr (rs76832527, [13, 31, 32]) and the recently described African-related p.Ser914* (rs60985508, [12]). Ancestrally shared PDVs p.Ile740Leu (rs74804606) and p.Ser914* (rs60985508) showed a higher prevalence in African patients (logistic regression models, p.Ile740Leu/rs74804606: Europeans *vs* Africans; odds ratio/OR = 0.04, 95% confident interval/CI = 0.005–0.30, *P*-value =0.02; p.Ser914*/rs60985508: OR = 0.01, 95% CI = 0.002–0.09, *P*-value = 2.03e−05).

### Common germline *ANO7* variants associated with African-ancestral PCa risk

Of the 17 *ANO7* variants represented within our 473 genotyped southern African PCa cases and 307 cancer-free controls (Supplementary Table S2), six identified PDVs were overlapped with our sequencing data (Table 1, Supplementary Fig. S2). While PDV p.Ile740Leu (rs74804606) was associated with PCa risk (Wilcoxon test, FDR = 0.03; Supplementary Table S2), the European-derived PCa-risk variant p.Ala759Thr (rs76832527) was rare in our study with no associated risk.

### Intercorrelation of germline *ANO7* variants

We investigated the correlation between germline *ANO7* PDVs or SVs, with known PCa-risk variants (Spearman’s test, FDRs = 0 to 2.84e−03, Table 2). Correlations identified exclusively in African patients involved four germline SVs while the correlation specific to European patients involve a PCa-risk synonymous variant rs62187431, whilst rs62187431 was also correlated with two germline SVs exclusively in the African patients (Supplementary Table S3). An ancestrally shared correlated pair was observed between two PDVs, p.Ile740Leu (rs74804606) and p.Ser914* (rs60985508), cooccurred in 29 Africans and a single European. The other correlated pair between PDVs, p.Asp789Leu (novel frameshift) and p.Asp789Val (rs527323541), cooccurred together in five African patients, which together truncated the Anoctamin 7 protein by 100 amino acids. As the less frequent variant only occurred in a subject when the more frequent variant was present, most were defined as inclusive correlated (IC) pairs (Supplementary Fig. S3). The IC pair p.Ala759Thr (rs76832527) and rs62187431 whose linkage disequilibrium (LD) was also reported in Asian patients [15] were in the same haplotype block with strong LD in European patients.

**Table 2:** Correlations between PDVs and other variants.

Pairs of correlated variants		Ethnicity	*ρ*^a^	FDR	IC^b^	Distance (kb)
p.Asp789Val	p.Asp789Leu (stop-gain)	African	1	0	Y	Adjacent
p.Tyr440Asn	g.21684_22027del	African	0.49	3.61E−06	Y	3.8
p.Arg578Cys	g.23653_23712del	African	0.49	3.61E−06	Y	4.9
p.Arg578Cys	g.4185_4328dup	African	0.49	3.61E−06	Y	24.3
p.Leu734Pro	g.27267_27392del	African	0.4	9.44E−04	Y	6.9
p.Ile740Leu	p.Ser914* (stop-gain)	African	0.37	2.84E−03	N	6.2
		European	1	0	Y	6.2
p.Ala759Thr	rs62187431	European	0.92	1.32E−23	Y	5.5

PDVs defined as variants with SIFT scores under 0.05 or PolyPhen scores greater than 0.446; Other variants including benign, tolerated or structural variants.

^a^Rho (Spearman’s correlation coefficient).

^b^Whether the correlated pair is an IC pair. Y for yes and N for no.

### *ANO7* germline PDVs linked with early-onset PCa

Correlating PCa measurements and *ANO7* variants using linear regression analyses with adjustments for the number of small germline variants and PCa-risk levels, we found African patients to present six years earlier at diagnosis (−6.42 years, 95% CI = −10.68 to −2.16, *P*-value = 0.003) if carrying three or more selected germline variants (PDVs and/or SVs), regardless of zygosity (Supplementary Fig. S4). Of these 15 African patients, 14 carried at least two germline PDVs and one carried two germline SVs, suggesting an accumulation of inherited PCa risk from selected *ANO7* variants. While none of our European patients presented with greater than two selected variants, it should be noted that *ANO7* rs77559646 has previously been reported to be associated with early-onset PCa in a Finnish study [11].

### *ANO7* as a putative cancer driver

Somatic variants, 23 small variants (Supplementary Table S4) and two inter-chromosomal fusion SVs (Supplementary Figs. S5 and S6), were biased towards high-risk tumours (Gleason score $\ge$4 + 3) derived from patients of predominantly African ancestry (8 out of 9). An African missense p.Phe79Leu (rs1217170132) was annotated with a deleterious impact on an alternative transcript (ENST00000451047), which has not been reported in population data or previous studies. Additionally, and novel to this study, we found *ANO7* to act as a fusion partner for oncogenic genes, namely G3BP Stress Granule Assembly Factor 1 (*G3BP1*) at 5q33.1 [33–35] and PTPRF interacting protein alpha 4 (*PPFIA4*) at 1q32.1 [36–38]

### Impact of *ANO7* PDVs on protein structure

**Fig. 2:**
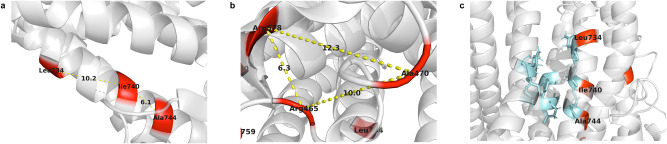
Closely distributed of PDVs on Anoctamin 7 protein. **a**, **b**, Two groups of closely distributed PDVs. **a** p.Ile740Leu (rs74804606) is close to p.Leu734Pro (novel) and p.Ala744Gly (rs773052325) with the structural distance of 10.2 Å and 6.1 Å, respectively. **b** PDVs p.Arg465Trp (rs145157097), p.Ala470Val (rs887541003) and p.Arg578Cys (rs111934267) are close to each other with labelled distance in Å unit. **c** Putative Ca^2+^ binding sites and adjacent PDVs. Putative Ca^2+^ binding sites are in blue, shown as sticks, which are residues p.Asn655, p.Asn656, p.Glu659, p.Asn735, p.Glu707, p.Glu 710, p.Glu739, and p.Asp743.

Of the 13 PDVs in WGS (Table 1, Supplementary Fig. S1), 11 clustered in the Calcium (Ca^2+^)-activated chloride channel (Supplementary Fig. S1), while a single PDV was located in an anoctamin dimer region. Two clustering of PDVs in proximity with distances <13 Å were predicted in the tertiary structure (Supplementary Fig. S7, Fig. 2a, b), with one clustering, p.Ile740Leu (rs74804606) and p.Ala744Gly (rs773052325), located adjacent to putative Ca^2+^ binding sites (Fig. 2c) originally identified in Anoctamin 1 protein [39].

**Fig. 3:**
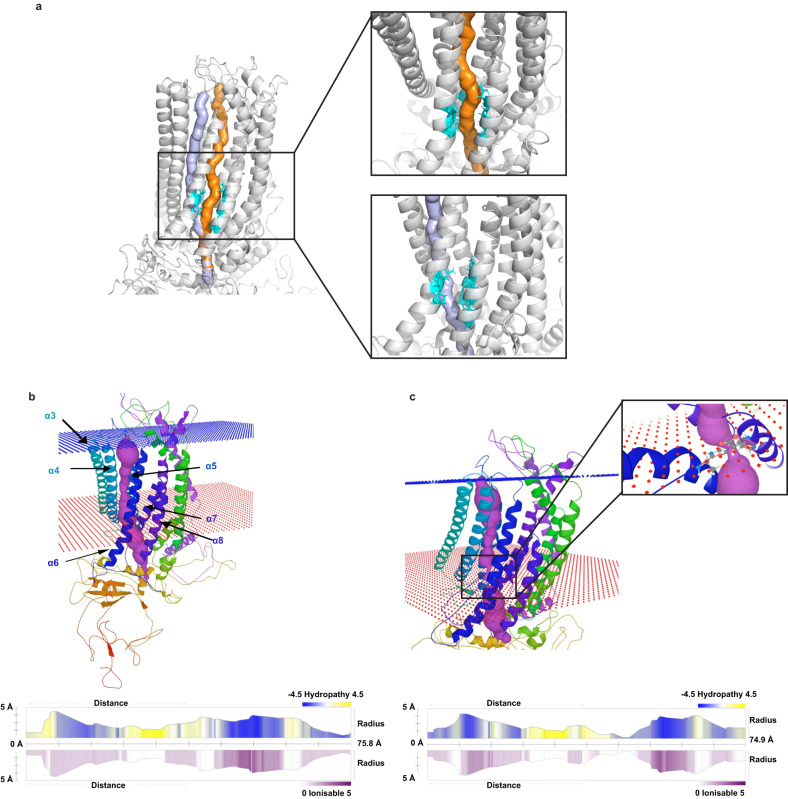
Ion conduction pore predicted in Anoctamin 7 protein. **a** Two possible pores with zoomed-in views of the Ca^2+^ binding region. Pores 1 and 2 are in orange and light purple, respectively. Putative Ca^2+^ binding sites are in cyan. **b** Top part: placement of Pore 2 among helices α4–8. Bottom part: The Y values of bar plots indicate radii of the pore. The top bar plot shows hydropathy with colours where blue indicates hydrophilicity and yellow indicates hydrophobicity. The bottom bar shows an ionisable capability, where the darker the purple, the easier to be ionisable. **c** Pore 2 identified in altered Anoctamin 7 with p.Ala470Val (rs887541003) and p.Ile740Leu (rs74804606). Top part: placement of pore 2 in altered protein. Bottom part: reduced bottleneck for Pore 2 in altered protein.

Two potential ion conduction pores were identified in the transmembrane domains (TMDs) and passed through putative Ca^2+^ binding sites (Fig. 3a). The two pores were close among helices α5–8 at the end connecting to the cytoplasm and apart at the other end where Pore 1 was circled by helices α5–9, while Pore 2 was tilted with helices α4–6 surrounded, the similar placement of ion conduction of Anoctamin 1 protein [39]. Their properties were similar that were hydrophobic and less ionisable in the central part near the Ca^2+^ binding sites and with radii larger than 1 Å within TMDs (bottlenecks, Pores 1 = 1.6 Å, Pores 2 = 1.3 Å, Supplementary Fig. S8). The result of pore identification changed with presence of PDVs, which could hinder the movement of ions and affect the interaction between Ca^2+^ and Anoctamin 7 protein. For a protein with p.Ala470Val (rs887541003) and p.Ile740Leu (rs74804606), Pore 1 was not identified, while Pore 2 was identified as narrower with 0.4 Å bottleneck radius and was less hydrophilic and ionisable at the centre of the pore above the bottleneck (Fig. 3b, c). The pore alteration could be the consequence of positional changes of residues 673–693, which were also observed when other PDVs were present (Supplementary Fig. S9).

## Discussion

Recently pinpointed as an African-relevant PCa-risk locus, here we performed a thorough investigation for the role of *ANO7* variants in PCa predisposition and aggressive disease in men from southern Africa, identifying numerous potential roles for *ANO7* in driving ancestrally-linked PCa health disparities. Firstly, we validated the African-related PCa-risk variant p.Ser914* (rs60985508) [12] in our study through co-occurrence with the African dominant p.Ile740Leu (rs74804606), which has previously been associated with estrone per androstenedione ratio in women with increased risk of breast cancer [40]. Conversely, the European-specific PCa-risk deleterious variant p.Ala759Thr (rs76832527, [13, 31, 32]) showed no significant difference in our African cohort. Besides the strong LD between p.Ala759Thr (rs76832527) and the PCa-risk variant rs62187431 in our European patients, which has been verified in an Asian study [15], the higher p.Ala759Thr (rs76832527) MAF suggests earlier divergence in Europeans. Taken together, these deleterious variants with distinct frequencies across ancestries may potentially account for the divergent PCa outcomes across ancestries.

Given that variation in conserved amino acid residues can potentially impact protein properties [41], we further examined for the possible impact of identified African-relevant PDVs on the *ANO7* protein that is known to be Ca^2+^ dependent for being either CACC or PLS [7]. A previous Anoctamin 1 study showed that co-occurrence of variants at residues 740, 759 and 775 (Anoctamin 7 equivalent residue positions) significantly decreased channel activity [42]. Our study shows that the impaired activity with the presence of PDVs is likely to be caused by a decrease in binding affinity and ion selectivity in proximity to Ca^2+^ binding sites and in ion conduction pores through the binding sites. Those affecting PDVs are either African-specific or with higher prevalence in African than European patients. Three PDVs, namely p.Leu734Pro (novel), p.Ile740Leu (rs74804606) and p.Ala744Gly (rs773052325), are located neighbouring to Ca^2+^ binding sites and one PDV p.Ala632Val (rs139066448) is within the re-entrant structure (residues 628–657) [7]. Additionally, the obstruction of predicted ion conduction paths was observed in proteins containing PDVs such as p.Ile740Leu (rs74804606). The changed pore properties include narrower bottlenecks in TMDs and differential hydrophilic and ionisable capabilities near binding sites. These impairment on *ANO7* protein may be relevant to the observed overexpression in malignant tumour cells [43].

Novel to this study, we identify *ANO7* as a potential oncogenic driver in African men, through the formation of gene fusions with the cancer-related genes *G3BP1* and *PPFIA4*, involved in androgen receptor (AR) [35] and mitogen-activated protein kinase (MAPK) signalling [44], respectively. The protein of *G3BP1* promotes PCa tumourigenesis by binding to a PCa-specific suppressor *SPOP* [40]. The *G3BP1-SPOP* bound ubiquitin activates AR signalling and upregulates *G3BP1* transcription [35], leading to overexpression of *G3BP1* in PCa tumour cells [33, 34] and further inhabitation of the tumour suppressor *SPOP* [35]. The second fusion gene partner *PPFIA4* has been observed to be overexpression in PCa patients having experienced biochemical relapse after radical prostatectomy [37]. The *PPFIA4* protein liprin-α4 is involved in the MAPK signalling pathway [44] which may cause castration-resistant PCa through AR pathway independence [45, 46], and has been proposed as a potential therapeutic target for several cancer types [47, 48]. Contradictorily, *PPFIA4* is a hypoxia-induced gene potentially stabilise cell-cell contacts [49] and may prevent invasion of PCa cells [38].

## Conclusions

In conclusion, the present study on *ANO7* variants has shown genetic differences between Africans and Europeans and correlations with PCa, indicating the role of ethnicity in the implication of genetic variants in PCa. The alterations of protein structure caused by *ANO7* variants may exert an impact on molecular function and may further promote tumourigenesis. These findings underline the possibility that *ANO7* variants are involved in an ancestrally-related multi-hit processes of carcinogenesis and emphasise the necessity of a future study of *ANO7* variation and clinical correlation in a larger sample size of African patients.

### Supplementary information

Supplementary File

Supplementary Table S5

Supplementary Table S8

## Data Availability

The data used in this study will be made available on request.
