# Supplementary material for: ANO7 African-ancestral genomic diversity and advanced prostate cancer
Source: Prostate Cancer Prostatic Dis. 2023 Sep 25;27(3):558–65. doi: 10.1038/s41391-023-00722-x (PMC11319200; doi:10.1038/s41391-023-00722-x)
Supplement: Supplementary file 1 — Supplementary File [file 41391_2023_722_MOESM1_ESM.pdf]

ANO7 African-Ancestral Genomic Diversity and Advanced Prostate Cancer

Jue Jiang, Pamela Soh, Shingai B.A. Mutambirwa, M.S. Riana Bornman, Christopher A. Haiman, Vanessa M. Hayes and Weerachai Jaratlerdsiri

Supplementary File

Supplementary Methods

Ethnic approvals and data preparation for the Exome array case-control study

The investigation of African-related Prostate cancer (PCa) causal variants was conducted on a case-control study of 798 South Africans, recruited as part of the Southern African Prostate Cancer Study (SAPCS) and both population and ancestrally matched to the African whole genome sequencing (WGS) data. Men were consented under the University of Pretoria Faculty of Health Sciences Research Ethics Committee (HREC) SAPCS approval #43/2010 (with US Federal wide assurance FWA00002567 and IRB00002235 IORG0001762). Genomic DNA was extracted from whole blood (Qiagen) and shipped to Australia under Republic of South Africa Department of Health Export Permit (National Health Act 2003; J1/2/4/2 #1/12) and University of Pretoria and University of Sydney Material Transfer Agreement (MTA). Genomic interrogation was performed under the St. Vincent's Sydney HREC study approval #SVH/15/227.

Annotation of short variants

The annotation of short variants was processed with the online tool SNPnexus (https://www.snp-nexus.org/v4/) (1). SNPnexus provides multiple tools and datasets using the publicly accessible Ensembl gene annotation system (Ensembl Variation 95). The database includes HapMap (Nov 2018 updated), 1000 Genomes (Nov 2018 updated), and gnomad v2.1(Mar 2019 updated). Predicted effects of single nucleotide variants (SNVs) were merged from two annotation tools, including Sorting Intolerant From Tolerant (SIFT, Jan 2019 updated) (2) and Polymorphism Phenotype (PolyPhen, Jan 2019 updated) (3). Predicted deleterious variants (PDVs) of the main transcript of *ANO7* EN single nucleotide variant ST00000274979 included deleterious variants predicted by SIFT and probably/possibly damaging variants predicted by PolyPhen, as well as indels with stop-gain/frameshift effects predicted with Ensembl (4). Minor allele frequency (MAF) of PDVs in African and European populations were obtained from online Allele Frequency Aggregator (ALFA) (5).

Sequence analysis

The sequence and phylogenetic analyses were processed using MEGA (v11) (1) where multiple algorithms and methods are available for each step. A total of 45 unique sequences of amino acid sequences were obtained by replacing the original transcript (ENST00000274979) with respectively germline missense variants for each of the 166 patients. Multiple sequence alignment of the 45 sequences was made using MUSCLE in MEGA (2). The best protein model estimated by PhyML (v 3.0) (3) was Jones-Taylor-Thornton (JTT) with evolutionary rates among sites invariable and following discrete Gamma distribution, which in turn was used for the construction of phylogenetic trees and estimation of pairwise genetic distance in MEGA. We used the neighbour-joining statistical method and bootstrap

values equal to 1,000 to construct a phylogenetic tree, which was only assessed for groupings due to low branch support.

Pairs of correlated variants and haplotype block analysis

Correlations between *ANO7* PDVs and other important variants, including germline structural variants (SVs) and previously reported PCa causal SNVs, were accessed using Spearman's rank correlation coefficient (ρ) from Stats package in R, which assumes no frequency distribution. Significantly correlated pairs were those with FDR<0.05 after *p*-value adjustment by multiple hypothesis correction using Rstatix package (v 0.7.0) (4).

Haplotype block analysis of SNVs within *ANO7* was conducted with Haploview (v 4.1) (5). Input data was prepared using VCFtools (v 0.1.14) (6) excluding multi-allelic variants, insertions, or deletions. Haploview calculated pairwise measures of LD between SNVs with MAF>0.001, and defined LD blocks under default confidence intervals where 95% of the SNVs were considered in strong LD (5). The strong LD of a pair was defined if the one-sided lower and upper 95% confidence bounds on D-prime were above 0.7 and 0.98, respectively (7).

Age at diagnosis and *ANO7* variants

The associations between age at diagnosis and selected *ANO7* variants was investigated with linear regression models using Stats package in R. Variables were examined using t-test with a threshold of significance at a *P*-value of 0.05. An indicator was made to indicate whether a patient carried more than two variants of PDVs and/or germline SVs. One African patient was filtered out for lacking age information and all the European patients were excluded as none presented with more than two selected variants. The analysis cohort (n=108, all African) consisted of 93 patients with <3 selected variants and 15 patients with ≥3 selected variants. As the linear regression model allows assessment of effects of multiple variables simultaneously, the best model was determined by the fitness of the model estimated by Akaike's Information Criterion (AIC) in stepwise selection. Variables in the best model included the indicator of whether having more than two selected variants, the total count of genome-wide short germline variants and PCa risk levels. Genome-wide tumour mutational burden (TMB) was tested but was not selected.

***ANO7* variants prevalence in ethnic groups**

The difference of allele frequency in different ethnic groups (African, n=109 and European, n=57) was compared for 13 PDVs using logistic regression models from Stats package in R. The logistic regression analyses model the probability of a binary discrete variable, so the genotype information was transformed into a binary variable where "0" means no alternate alleles and "1" means otherwise. The significant level was tested using t-test, and corrected by false discovery rate (FDR) using Rstatix package (v 0.7.0) in R (v 4.1.3;). Significantly differential MAFs were defined by FDR<0.05.

Pores identified in all the sequences containing PDV p.Ile740Leu

Ten unique sequences across the study cohort contain p.Ile740Leu and other missense variants if co-occur in a patient. Identified pores in the ten sequenced were classified into Pores 1-2 if showing the same placement of pores identified in the original protein, and into two new pores named as Pores 3-4, listed in **Table S7** and presented in **Figure S13-S17.**

Pores 1-2 with normal radius were both identified in Seq 39 that contains p.Ile740Leu and p.Asp156Glu while Pore 2 with normal radius was found in Seq 43 that contains p.Ile740Leu and p.Asp70Asn. Save for the above two altered proteins, Pores 1-2 with narrower bottlenecks were observed in altered proteins (Seq 28 for narrower Pore 1, and Seqs 9, 28, and 31 for narrower Pore 2), while neither of the Pores 1-2 were identified in the other four altered proteins (Seqs 18-20, 22).

Two new pores were identified repeatedly in altered proteins and both bypassed the putative Ca2+ binding sites. Pore 3 is the one with one end among at helices α1-2, 9 and the other end among α5-9 (Figure S12a), identified in two proteins (Seqs 12 and 18). Pore 4 is among helices α 5,7, 9 for one end and among α 5-7, 9 for the other end (Figure S12b), reported in three proteins (Seqs 20, 22, and 28). Protein predicted with Seq 19 only showed a broken pore.

References

1. Stecher G, Tamura K, Kumar S. Molecular evolutionary genetics analysis (MEGA) for macOS. Molecular biology and evolution. 2020;37(4):1237-9.
2. Edgar RC. MUSCLE: a multiple sequence alignment method with reduced time and space complexity. BMC bioinformatics. 2004;5(1):1-19.
3. Guindon S, Dufayard J-F, Lefort V, Anisimova M, Hordijk W, Gascuel O. New algorithms and methods to estimate maximum-likelihood phylogenies: assessing the performance of PhyML 3.0. Systematic biology. 2010;59(3):307-21.
4. Kassambara A. rstatix: Pipe-friendly framework for basic statistical tests. Accessed: Feb. 09, 2022. 2021.
5. Barrett JC, Fry B, Maller J, Daly MJ. Haploview: analysis and visualization of LD and haplotype maps. Bioinformatics. 2005;21(2):263-5.
6. Danecek P, Auton A, Abecasis G, Albers CA, Banks E, DePristo MA, et al. The variant call format and VCFtools. Bioinformatics. 2011;27(15):2156-8.
7. Gabriel SB, Schaffner SF, Nguyen H, Moore JM, Roy J, Blumenstiel B, et al. The structure of haplotype blocks in the human genome. science. 2002;296(5576):2225-9.

Supplementary Tables
Tables S5 and S8, sperate excel files.

Table S1. Demographic and clinical information for the sequencing cohort studied

Ancestry	Sample size (n)	Country (n)		Median age (years, range)	Risk level (n)[a]		Median PSA (range)	
		South Africa	Australia		Low-risk	High-risk	Low-risk	High-risk
African	109	109	0	68.00 (45-99)	27	82	34.7 (7-194)	81.9 (4.3-4841)
European	57	4	53	63 (46-72)	7	50	7.3 (3.5-11)	8.2 (3.5-31.8)
Total	166	113	53	65.50 (45-99)	34	132	24.7 (3.5-4841)	

[a] Low-risk, Gleason score <4+3; High-risk, Gleason score ≥ 4+3

Table S2. Results of risk associations on 17 SNPs identified in exome array data

Protein change (NP001001891)	rsID	Major allele	Minor allele	PDV status[b]	Case (n=)	Control (n=)	MAF in case (%)	MAF in control (%)	*P*-value	FDR	Significance
p.Arg79Ser	rs150023062	G	C	No	473	307	0.11	0.33	0.333	1	ns
p.Asp156Glu	rs78972598	C	A	No	473	307	3.49	3.91	0.512	1	ns
p.Ala170Thr	rs141499501	G	A	No	473	307	0.00	0.16	0.215	1	ns
p.Gly242Arg	rs144166359	G	A	Yes (shared with WGS)	473	307	0.42	0.00	0.107	1	ns
p.Arg336His	rs201506858	G	A	Yes (array specific)	473	307	0.11	0.33	0.333	1	ns
p.Ala360Val	rs111978925	C	T	Yes (array specific)	473	307	0.11	0.00	0.422	1	ns
p.Cys397Tyr	rs145388383	G	A	Yes (array specific)	473	307	0.11	0.65	0.0624	0.936	ns
p.Tyr440Asn	rs147670958	T	A	Yes (shared with WGS)	473	306	1.16	0.82	0.507	1	ns
p.Ala494Val	rs57677160	C	T	No	466	303	5.79	5.28	0.808	1	ns
p.Arg578Cys	rs111934267	C	T	Yes (shared with WGS)	473	307	3.70	1.95	0.0455	0.728	ns
p.Val604Ile	rs111600763	G	A	No	473	307	2.01	3.09	0.169	1	ns
p.Arg612Arg	rs111624461	G	A	No	473	307	2.01	2.12	0.947	1	ns
p.Ala632Val	rs139066448	C	T	Yes (shared with WGS)	473	307	0.11	0.00	0.422	1	ns
p.Ile740Leu	rs74804606	A	C	Yes (shared with WGS)	473	306	21.56	15.36	0.00179	0.03043	*
p.Ala759Thr	rs76832527	G	A	Yes (shared with WGS)	473	307	0.11	0.16	0.759	1	ns
p.Glu912Lys	rs7590653	G	A	No	473	307	16.81	15.80	0.816	1	ns
intronic[a]	rs199829153	G	A	No	473	307	0.21	0.16	0.832	1	ns

[a]the SNP is in intronic region for main transcript of ANO7 ENST00000274979
[b]If being a PDV, variants will also be included in Table1

Table S3. Intercorrelations between germline structural variants (SVs) and risk variants.

Ethnicity	Paris of correlated variants		ρ[a]	FDR[b]	IC[c]	Distance (kb)
African	g.29592_29657del[d]	g.34404_34664del	0.39	9.44e-04	N	4.7
African	g.29592_29657del	rs62187431	1	0	Y	0.7
African	g.34404_34664del	rs62187431	0.39	9.44e-04	N	5.5

[a] rho, Spearman's correlation coefficient.
[b] false discovery rate (FDR).
[c] IC is short for inclusive correlated. Y is for inclusive correlated pairs and N is for non-inclusive correlated pairs.
[d] Genomic changes compared to NG029845.

Table S4. Somatic short variants identified in WGS data (n=166)

Chromosome	Position	Reference	Alteration	Genotype	Sample ID	Country	Ethnicity
chr2	241229784	G	GGCCCCCCCCCCCCCC	0/1	12103	Australia	European
chr2	241213160	T	G	0/1	SMU050	South Africa	African
chr2	241224600	C	T	0/1	SMU089	South Africa	African
chr2	241212509	C	A	0/1	SMU104	South Africa	African
chr2	241216852	G	C	0/1	UP2039	South Africa	African
chr2	241238248	G	A	0/1	UP2099	South Africa	African
chr2	241203105	C	T	0/1	UP2113	South Africa	African
chr2	241222172	T	C	0/1	UP2113	South Africa	African
chr2	241230726	G	A	0/1	UP2113	South Africa	African
chr2	241190868	C	T	0/1	UP2330	South Africa	African
chr2	241195874	C	A	0/1	UP2330	South Africa	African
chr2	241207888	T	G	0/1	UP2330	South Africa	African
chr2	241217082	C	T	0/1	UP2330	South Africa	African
chr2	241217847	T	C	0/1	UP2330	South Africa	African
chr2	241218541	TG	T	0/1	UP2330	South Africa	African
chr2	241220134	C	A	0/1	UP2330	South Africa	African
chr2	241221671	G	T	0/1	UP2330	South Africa	African
chr2	241222451	C	T	0/1	UP2330	South Africa	African
chr2	241222543	AT	A	0/1	UP2330	South Africa	African
chr2	241224346	G	A	0/1	UP2330	South Africa	African
chr2	241226831	T	C	0/1	UP2330	South Africa	African
chr2	241231960	C	T	0/1	UP2330	South Africa	African
chr2	241235400	G	A	0/1	UP2330	South Africa	African

Table S5. Haploview results of African and European WGS data.
Table in the attached excel file.

Table S6. Alpha helices and residues of protein ANO7

Alpha α helices	Residue start	Residue end
1	343	375
2	407	450
3	492	530
4	537	573
5	581	610
6	636	669
7	705	721
8	727	747
9	764	784
9 extend	790	796
10	842	869
10 extend	879	896

Table S7. Ten sequences that contains I740L and pores identified in the respective predicted proteins

No. Sequence	Missenses contained	Pore identification	Condition of the pore
Seq 9	p.Ile740Leu, p.Arg578Cys,	Pore 2	Narrower bottleneck (0.4Å, **Figure S14c**)
Seq 12	p.Ile740Leu, p.Val604Ile	Pore 3	Normal (**Figure S15a**)
Seq 18	p.Ile740Leu	Pore 3	Normal (**Figure S15b**)
Seq 19	p.Ile740Leu, p.Glu912Lys	A unique pore	Broken (**Figure S17a**)
Seq 20	p.Ile740Leu, p.Asp789Val	Pore 4	Normal (**Figure S16a**)
		Pore 4 alike	Broken (**Figure S17b**)
		A unique pore	Broken (**Figure S17c**)
Seq 22	p.Ile740Leu, p.Ala632Val	Pore4	Normal (**Figure S16b**)
		A unique pore	Broken (**Figure S17d**)
Seq 28	p.Ile740Leu, p.Ala494Val	Pore 4	Normal (**Figure S16c**)
		Pore 4 alike	Broken (**Figure S17e**)
		Pore 1	Narrower bottleneck (0.5Å) (**Figure S13b**)
		Pore 2	Narrower bottleneck (0.1Å, **Figure S14d**)
Seq 31	p.Ile740Leu, p.Ala470Val	Pore 2	Narrower bottleneck (0.4Å, **Figure S14e**)
Seq 39	p.Ile740Leu, p.Asp156Glu	pore 1	Normal (**Figure S13a**)
		pore 2	Normal (**Figure S14a**)
Seq 43	p.Ile740Leu, p.Asp70Asn	pore 2	Normal (**Figure S14b**)

Table S8. Information of samples for WGS study.
Table in the attached excel file.

Supplementary Figures

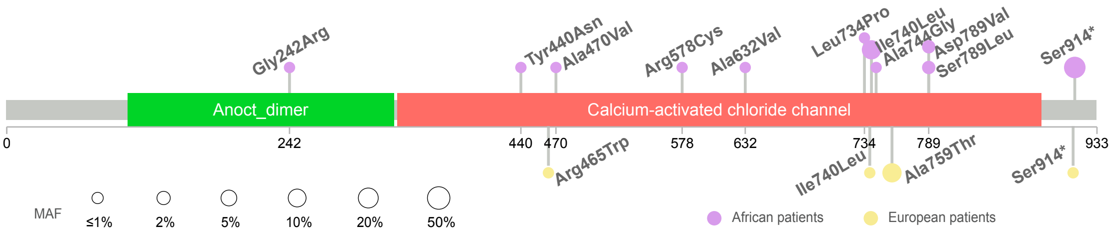

Figure S1. Minor allele frequencies (MAFs) of *ANO7* predicted deleterious variants (PDVs) in African (n=109) and European (n=57) prostate cancer (PCa) patients. Purple circles represent the MAF of PDVs in African patients while yellow circles represent the MAF in European patients.

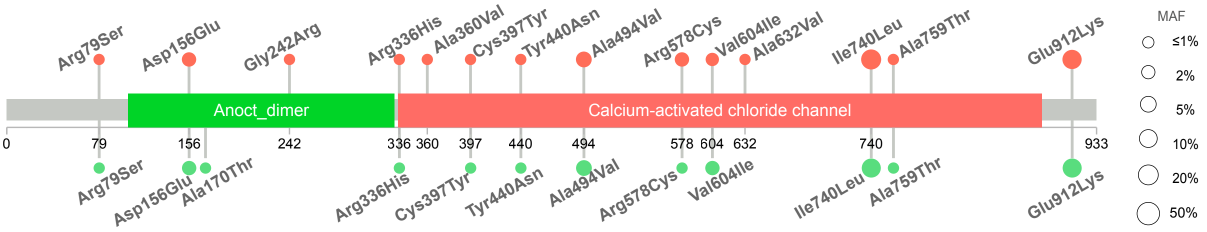

Figure S2. MAFs of 17 *ANO7* array SNPs in cancer cases (n=473) and controls (n=307). Red circles represent the MAF in cases while green circles represent the MAF in controls. Two SNPs (rs111624461 and rs199829153) were excluded from the lollipop plot due to no protein changes observed.

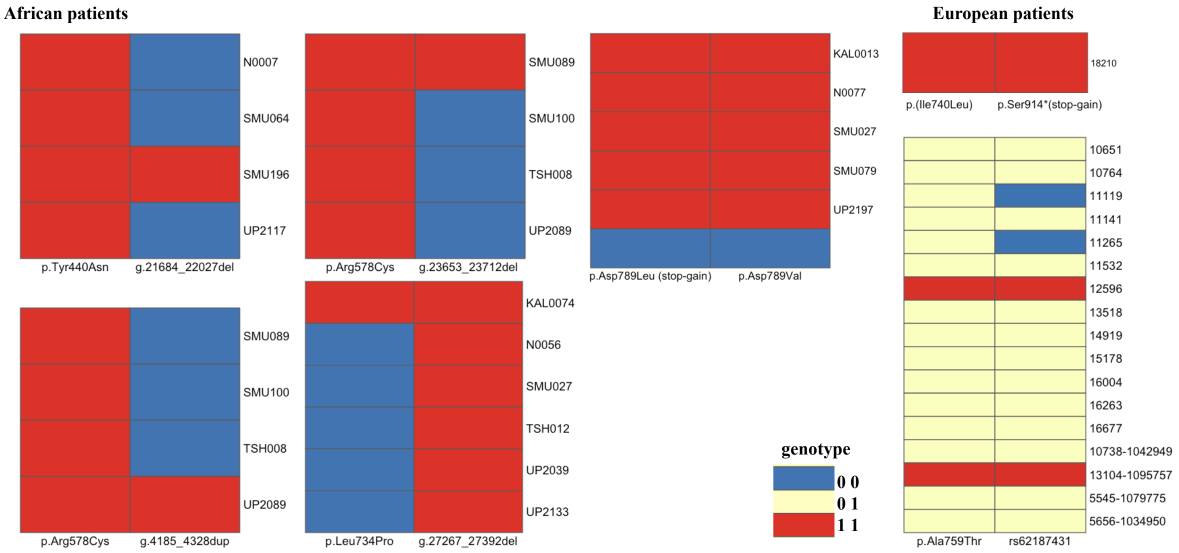

Figure S3. Genotypes of six inclusive correlated pairs in African and European patients. Rows are for the carriers of variants with sample IDs listed, and columns are for the two correlated variant. Colours represent genotypes of variants. Patients carrying no selected variants are in blue, carrying heterozygous variants are in yellow, and carrying homozygous variants are in red.

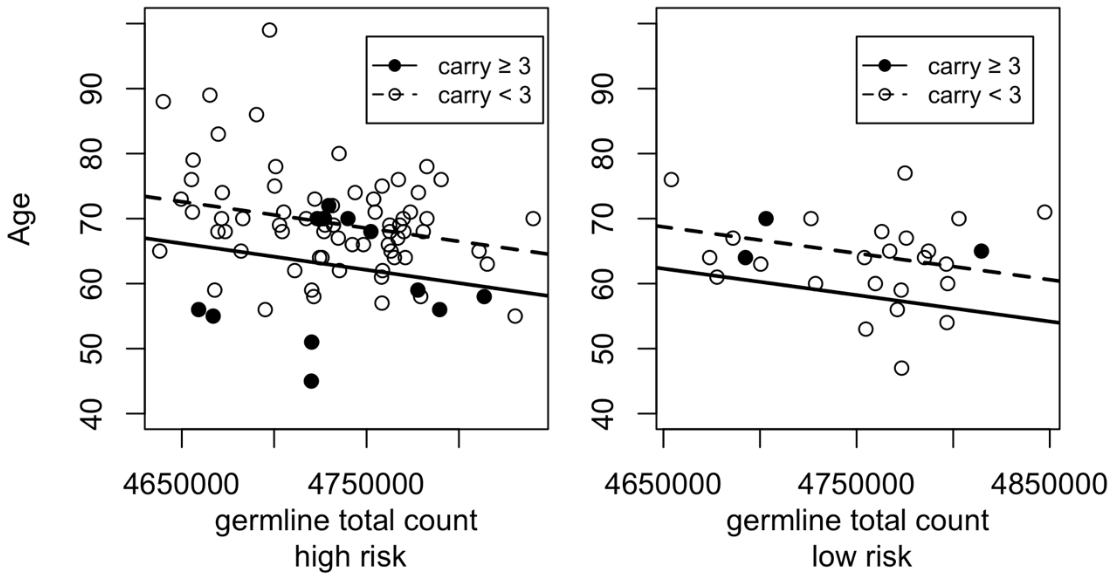

Figure S4. Age and total number of genome-wide germline short variants within African patients. The black dots represent African patients who had more than two selected variants (germline SVs and PDVs, n=15) and the white dots represent African patients with zero or one selected variant (n=93). *ANO7* germline SVs were counted when spanning part of or the whole region of *ANO7* gene. The effect of *ANO7* selected variants was adjusted with two confounding factors, including total number of genome-wide short germline variants (-4.07e-5, 95% CI=-7.21e-5 – -9.23e-6, *P*-value=0.01) and the risk of prostate cancer (-3.87, 95% CI=-7.34 – -0.4, *P*-value=0.03).

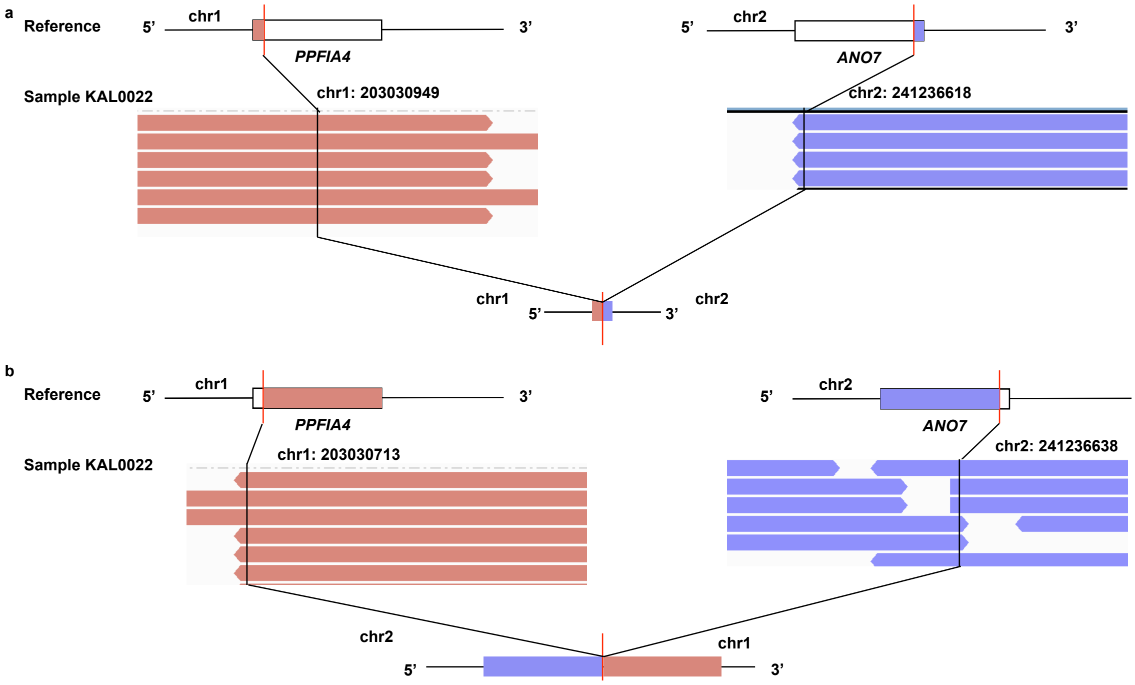

Figure S5. ***PPFIA4-ANO7* fusions.** The fusions between *PPFIA4* on chr1 and *ANO7* on chr2 was reported in two SV fusion events in sample KAL0022 at positions noted in red vertical lines. **a,** 3' end of *PPFIA4* at chr1: 203030949 was connected to 5'end of *ANO7* at chr2: 241236618. **b,** 5' end of *PPFIA4* at chr1: 203030713 fused with 3'end of *ANO7* at chr2: 241236638.

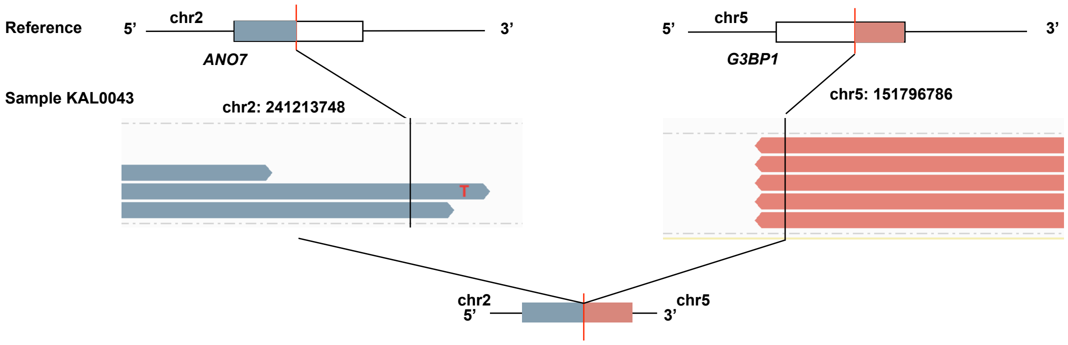

Figure S6. ***ANO7- G3BP1* fusions.** The fusions between *ANO7* on chr2 and *G3BP1* on chr5 was reported in sample KAL0043 with positions noted in red vertical lines. **a,** 3' end of *ANO7* at chr2: 241213748 was connected with 5'end of *G3BP1* at chr5: 151796786.

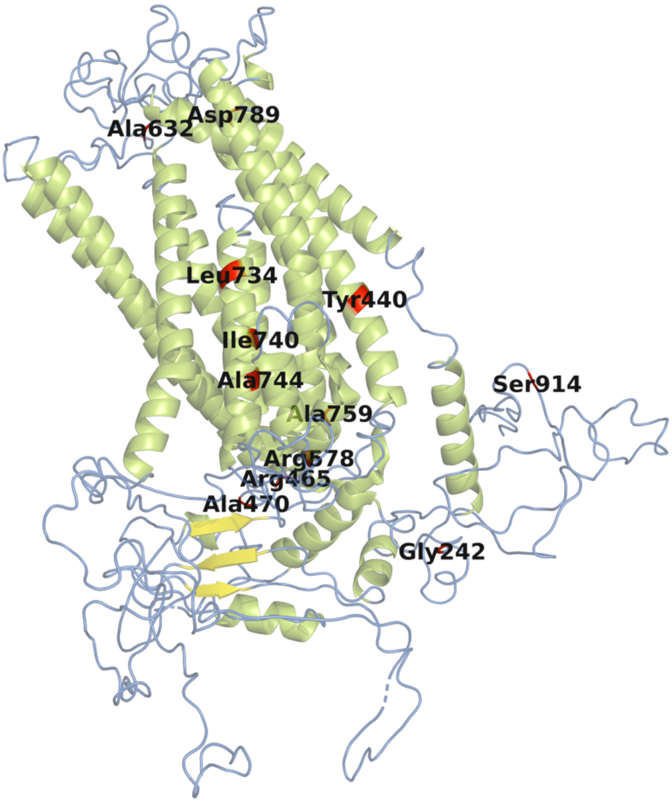

Figure S7. Distribution of PDVs (red) on the tertiary structure of Anoctamin 7 protein. The α helices are in green and β sheets are in yellow.

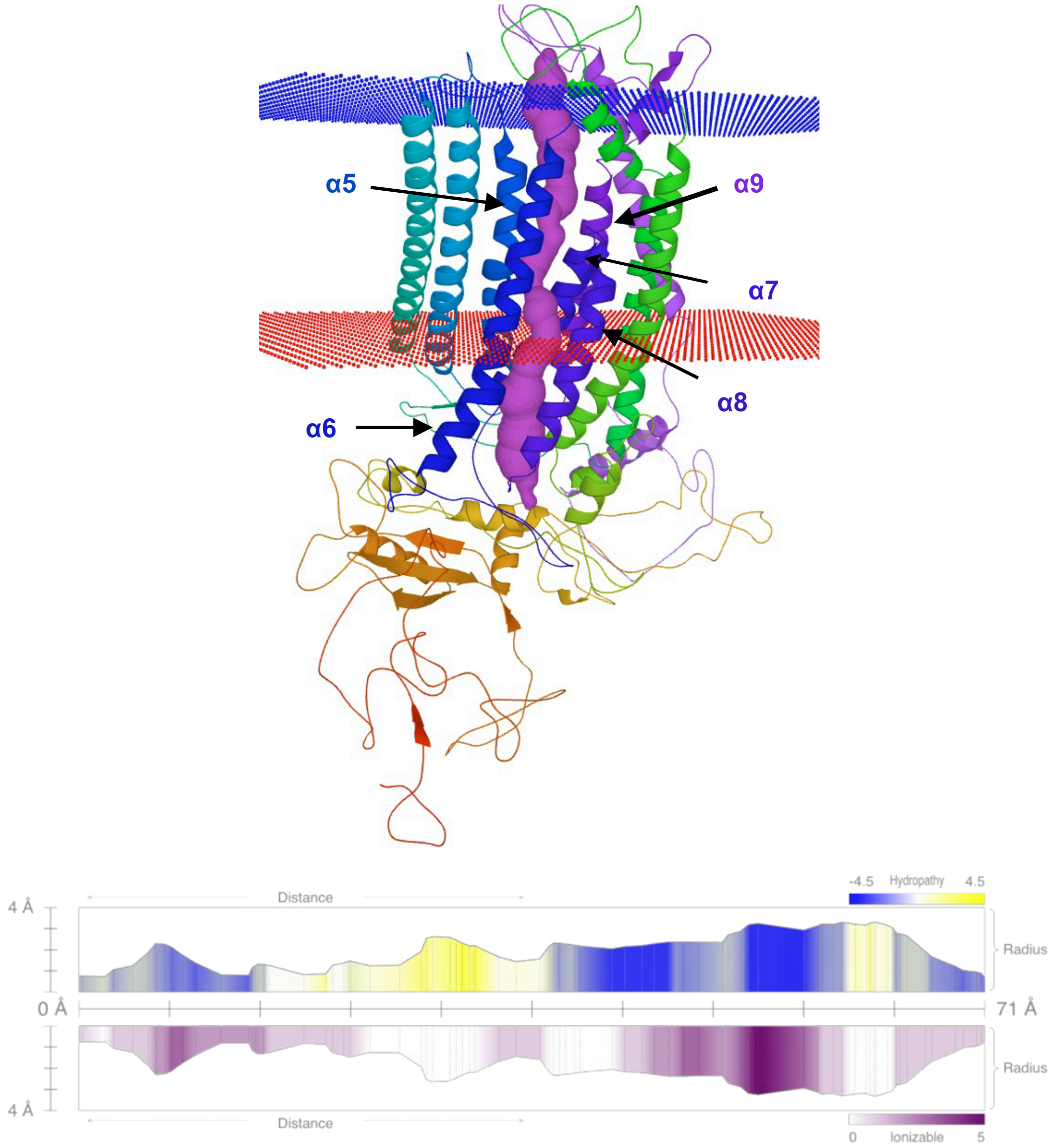

Figure S8. Ion conduction of Pore 1 predicted in Anoctamin 7 protein. The top part shows placement of Pore 1 is among helices α5-9. Transmembrane domains were between blue and red nets. The bottom part shows the properties of Pore 1. The Y values of bar plots indicate radii of the pore. The top bar plot shows hydropathy with colours where blue indicates hydrophilicity and yellow indicates hydrophobicity. The bottom bar shows ionisable capability, where the darker the purple, the easier to be ionisable.

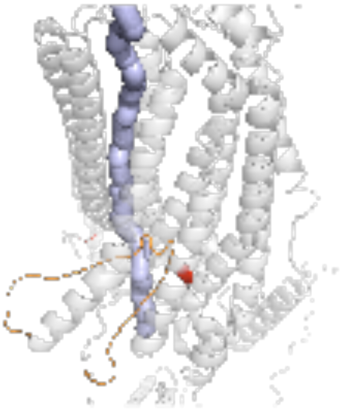

Figure S9. Alterations of Pore 2 identified in Anoctamin 7 with p.Ala470Val and p.Ile740Leu. Residues 673-694 are in orange. Pore 2 is in purple. PDVs are in red.

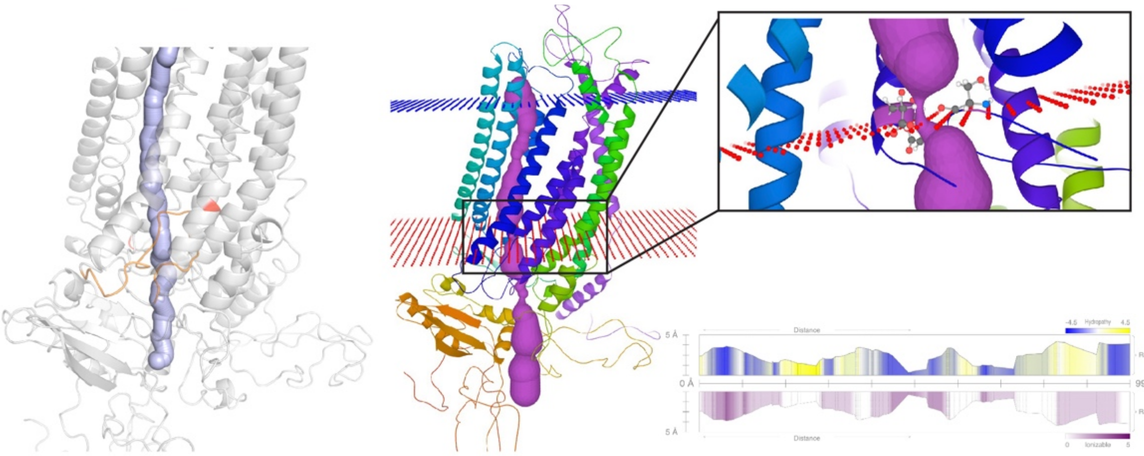

Figure S10. Alterations of Pore 2 in Anoctamin 7 with p.Ile740Leu and p.Arg578Cys. Left, residues 673-694 are in orange. Pore 2 is in purple. PDVs are in red. Middle and right top, reduced bottleneck is shown. Right bottom, characteristics predicted for Pore 2 in the altered protein. More detailed configurations are described in Figure S8.

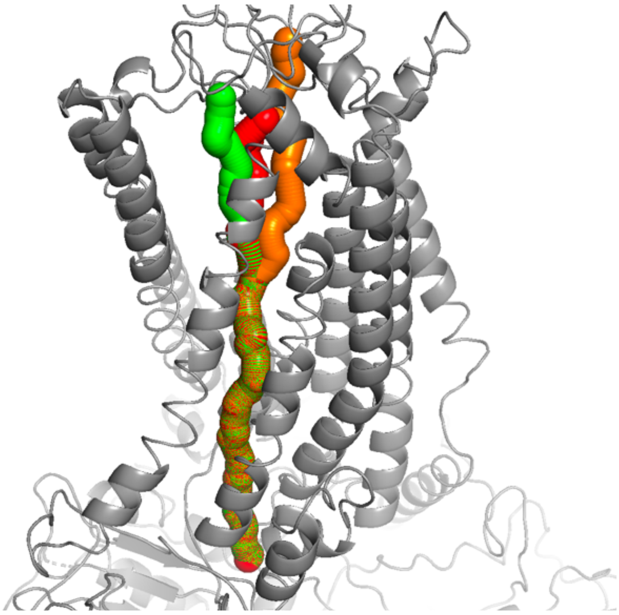

Figure S11 An example of a reproducible model of Pore 1. Three pores in green, red and orange colours are placed in the same group of helices α5-9, but divergent to each other within the top compartment of the model.

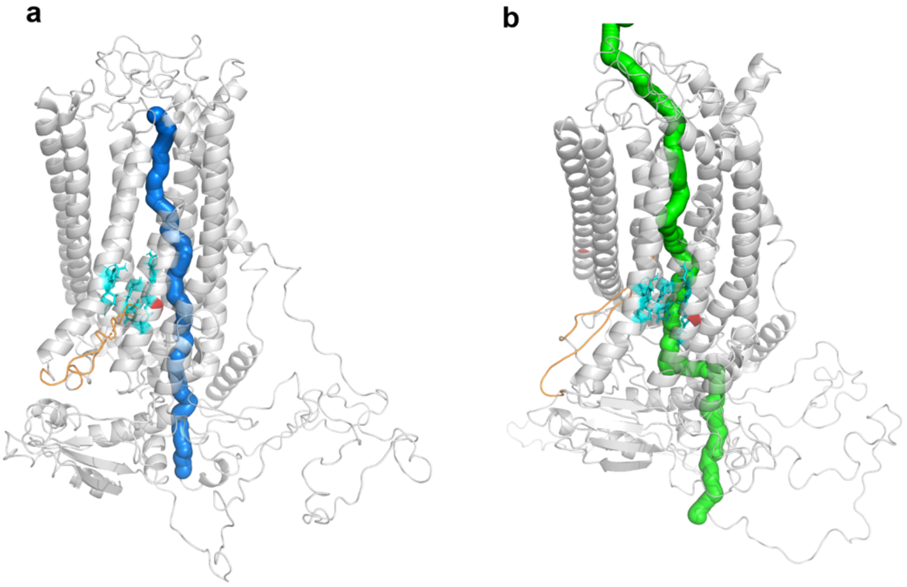

Figure S12. Pores that are commonly identified in altered proteins. a, Pore 3 in marine is shown in an altered protein that contains a PDV p.Ile740Leu in red and **b,** Pore 4 in green is shown in an altered protein that contains PDVs p.Ile740Leu and p.Ala494Val in red. Putative Ca^{2+} binding sites are in cyan, shown as sticks. Residues 673-694 that change positions are in orange.

a

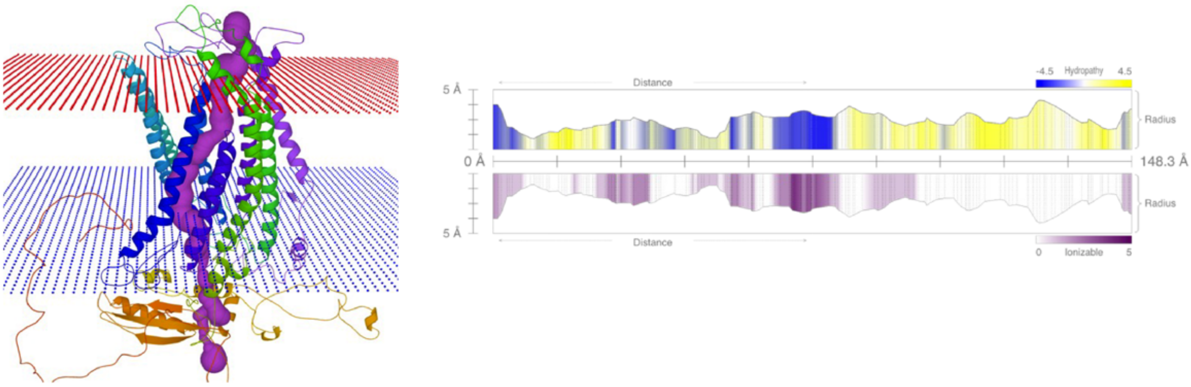

b

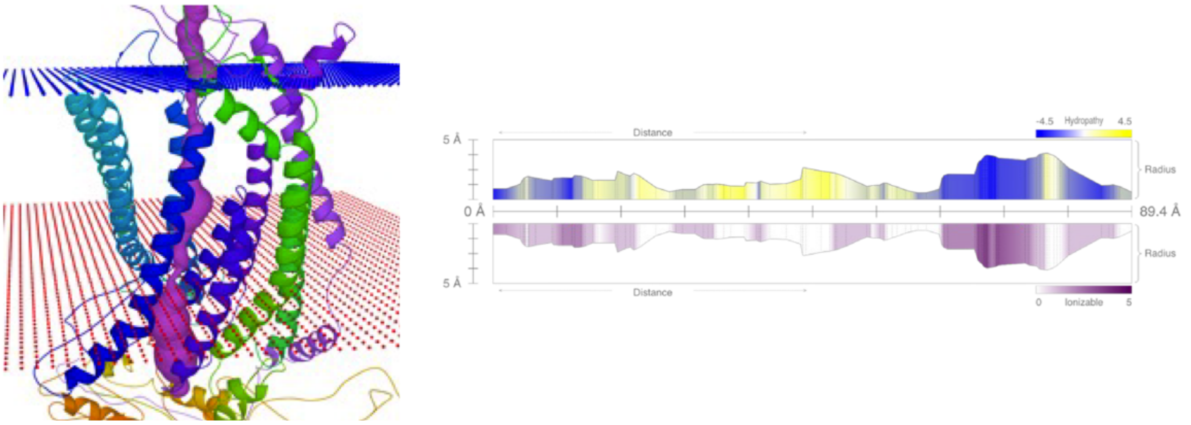

Figure S13. Pore 1 identified in altered proteins. a, Pore 1 for Seq 39 that is normal. **b,** Pore 1 (0.5Å) for Seq 28 that is narrower. The characteristics of respective pores are on the right plots. More detailed configurations are described in Figure S8.

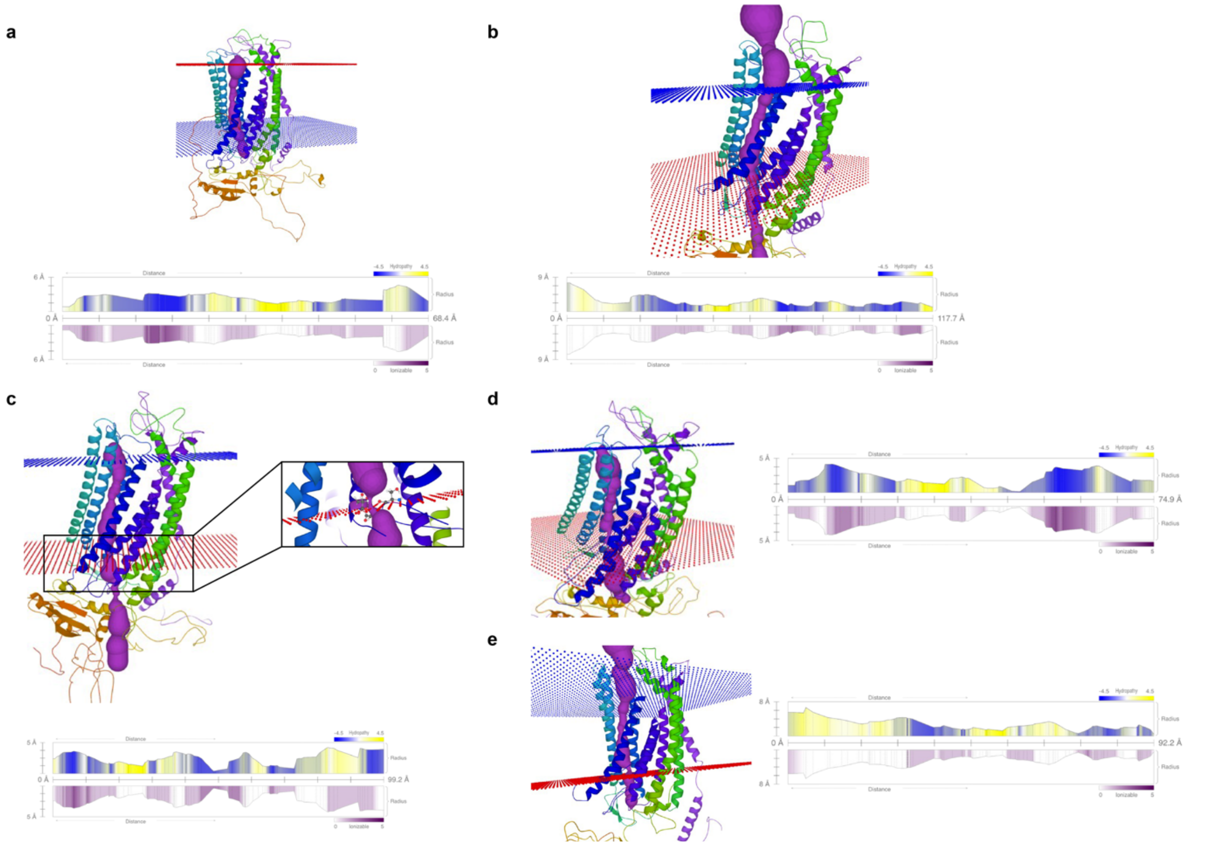

Figure S14. Pore2 identified in altered proteins. Pore 2 that is normal is shown for **a**, Seq 39 and **b**, Seq 43. Pore 2 that is narrower is shown for **c,** Seq 9 (0.4Å); **d,** Seq 28 (0.1Å); and **e,** Seq 31 (0.4Å). More detailed configurations are described in Figure S8.

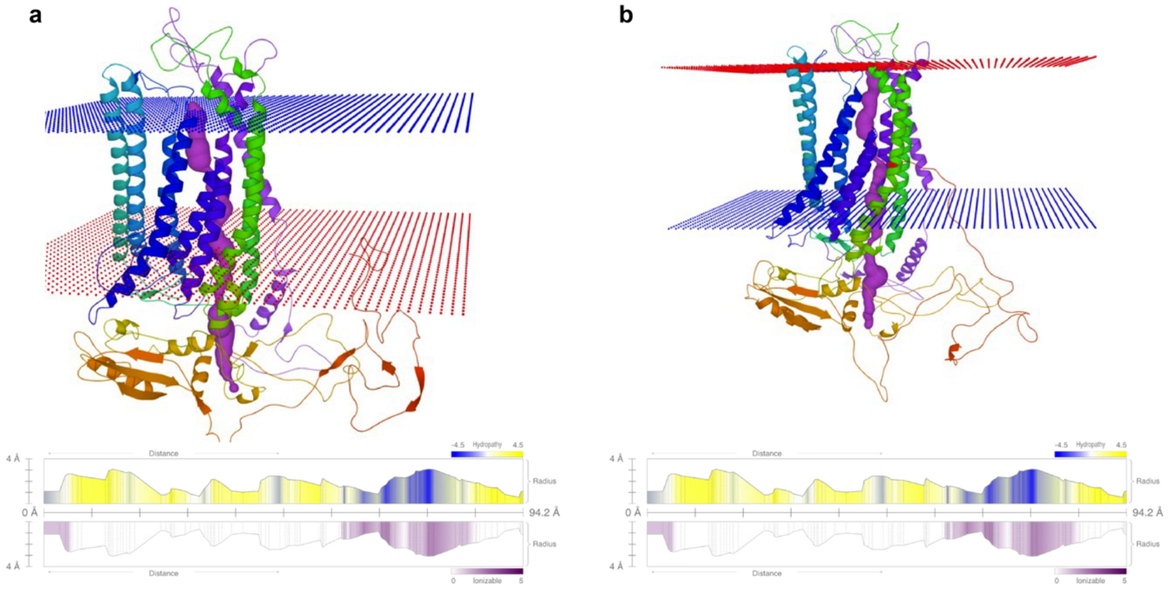

Figure S15. Pore 3 identified in proteins. a, Pore 3 for Seq 12 and **b,** Seq 18. More detailed configurations are described in Figure S8.

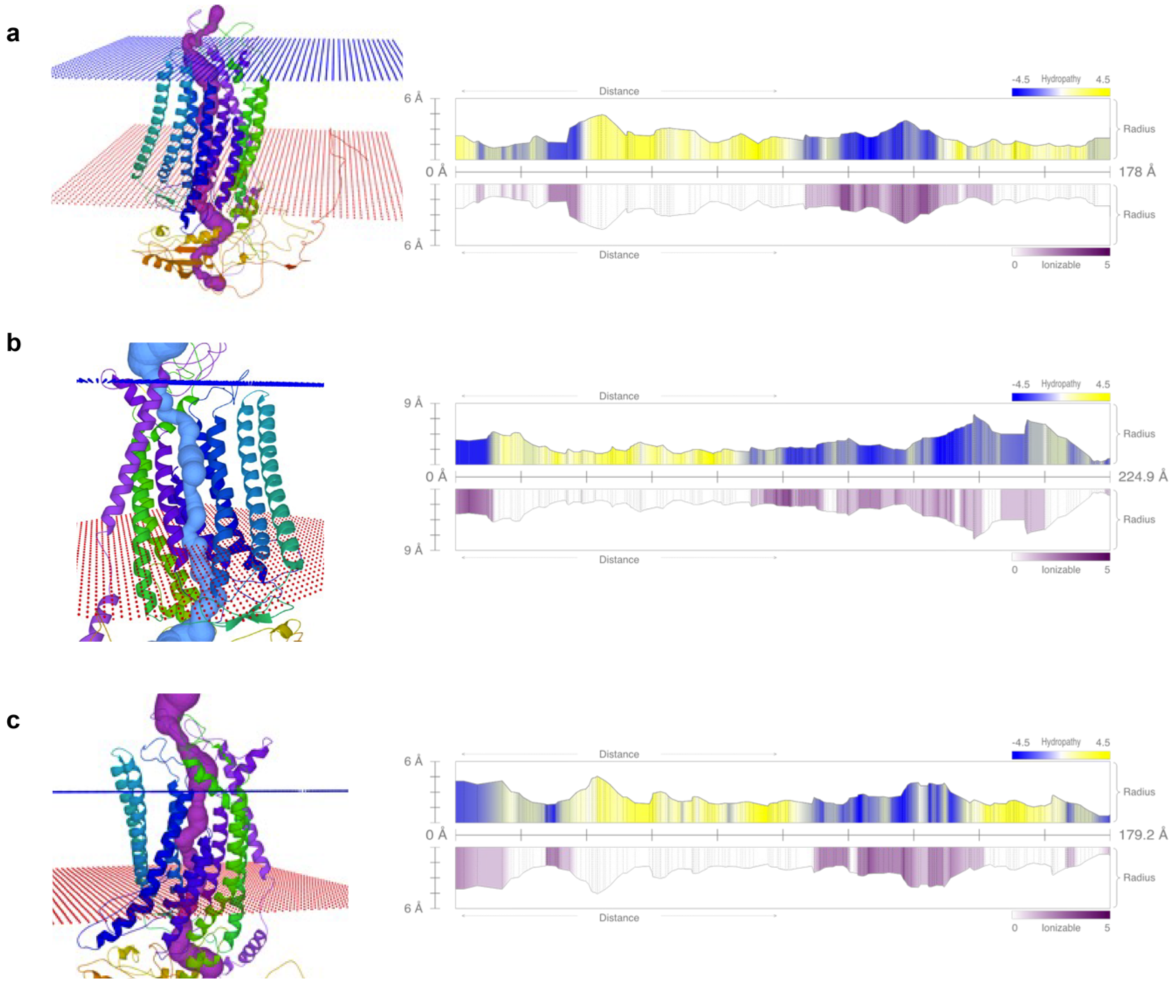

Figure S16. Pore 4 identified in proteins. a, Pore 4 for Seq 20; **b,** Seq 22; and **c**, Seq 28. More detailed configurations are described in Figure S8.

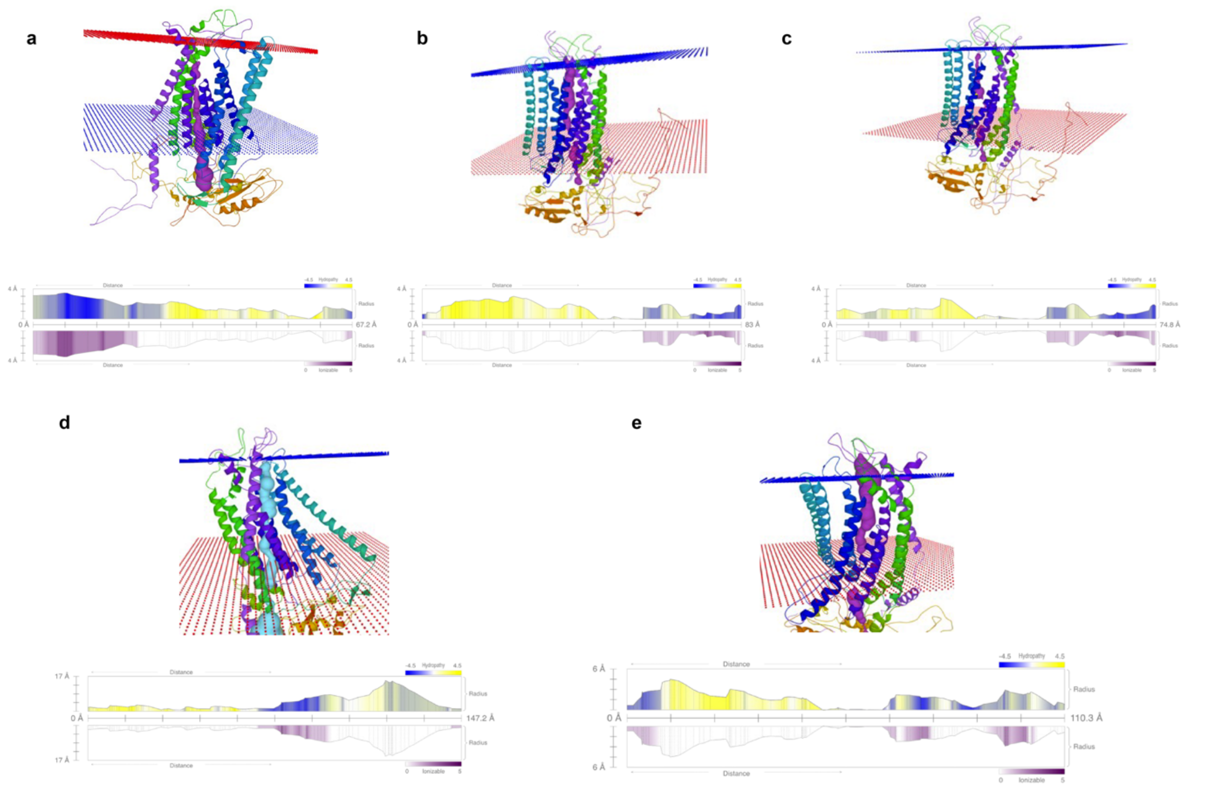

Figure S17. Positions and characteristics of broken pores. a, the pore broken in Seq 19. **b,** the pore identified in Seq 20 (Pore 4-like). It is broken when passing through helix α7. **c**, another broken pore identified in Seq 20. **d**, the pore broken in Seq 22. **e**, the pore identified in Seq 28 (Pore 4-like). It is broken when passing through helix α7. More detailed configurations are described in Figure S8.
